# Supplementary figures and images for: Morphological Alterations and Stress Protein Variations in Lung Biopsies Obtained from Autopsies of COVID-19 Subjects
Source: Cells. 2021 Nov 12;10(11):3136. doi: 10.3390/cells10113136 (PMC8623438; doi:10.3390/cells10113136)

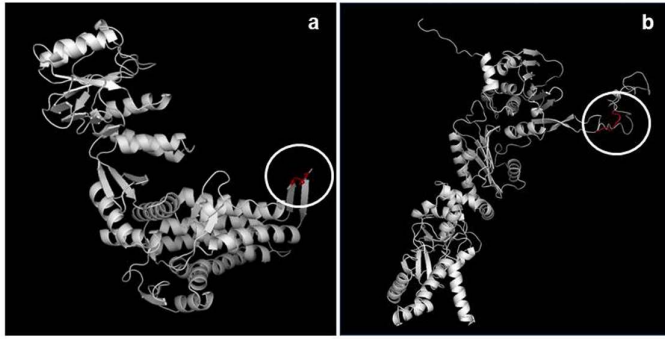

Figure S1: Three-dimensional model of human Hsp60 and Hsp90 monomer

Supplement: Supplementary file 1 [file cells-10-03136-s001.zip › cells-1448252-supplementary.pdf]
